# Stereochemical errors and their implications for molecular dynamics simulations

**DOI:** 10.1186/1471-2105-12-190

**Published:** 2011-05-23

**Authors:** Eduard Schreiner, Leonardo G Trabuco, Peter L Freddolino, Klaus Schulten

**Affiliations:** 1 Beckman Institute for Advanced Science Technology, University of Illinois at Urbana-Champaign, Urbana, IL 61801, USA; 2 CellNetworks, University of Heidelberg, 69120 Heidelberg, Germany; 3 Lewis-Sigler Institute for Integrative Genomics, Princeton University, Princeton, NJ 08544, USA; 4 Department of Physics and Beckman Institute for Advanced Science and Technology, University of Illinois at Urbana-Champaign, Urbana, IL 61801, USA

## Abstract

**Background:**

Biological molecules are often asymmetric with respect to stereochemistry, and correct stereochemistry is essential to their function. Molecular dynamics simulations of biomolecules have increasingly become an integral part of biophysical research. However, stereochemical errors in biomolecular structures can have a dramatic impact on the results of simulations.

**Results:**

Here we illustrate the effects that chirality and peptide bond configuration flips may have on the secondary structure of proteins throughout a simulation. We also analyze the most common sources of stereochemical errors in biomolecular structures and present software tools to identify, correct, and prevent stereochemical errors in molecular dynamics simulations of biomolecules.

**Conclusions:**

Use of the tools presented here should become a standard step in the preparation of biomolecular simulations and in the generation of predicted structural models for proteins and nucleic acids.

## Background

**Figure 1:**
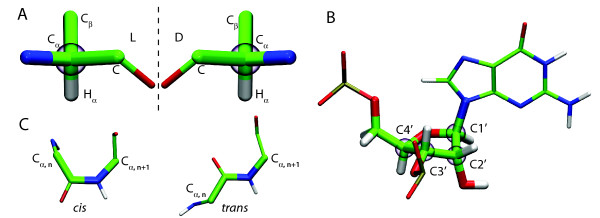
**Selected stereochemical parameters of amino acids and nucleosides**. (A) Chirality configuration at C*_α _*of an amino acid; (B) chiral centers at the ribose moiety of guanosine monophosphate; (C) *cis *and *trans *isomers of a peptide group. Carbon atoms are shown in green, nitrogen in blue, oxygen in red, and hydrogen in white. Chiral centers are surrounded by a transparent purple sphere.

Biomolecules often feature asymmetries in stereochemistry. Many biologically active molecules are chiral, i.e., they exist in two forms, called enantiomers, which are non-superimposable mirror images of each other. Of particular relevance to biological compounds is the carbon atom as a chiral center: a carbon atom is chiral if it carries four nonequivalent substituents. Thus, all amino acids save glycine have at least one chiral center at C*_α _*(see Figure 1A). Threonine and isoleucine have an additional chiral center at C*_β_*. Interestingly, only one of the two enantiomers is widely used in nature: according to the D-/L-naming convention, most naturally occurring amino acids are found in the L-configuration. Note, however, that D-amino acids do occur in biology, e.g., in cell walls of bacteria [1-3]. Nucleic acids also have chiral centers. For example, in DNA the atoms C1', C3', and C4' of the sugar moiety are chiral, while in RNA the presence of an additional OH group renders also C2' of the ribose chiral (see Figure 1B). Although the origin of the homochirality is not understood, the asymmetry due to preferential use of one enantiomer in biological systems has wide consequences. In particular, recognition processes of chiral molecules are impacted as can be demonstrated, e.g., by the different smell of D- and L-carvone or by the inhibition of proteases by D-amino acids [4]. On the level of protein structure, the occurrence of D-amino acids in an L-amino-acid environment is known to disrupt secondary structure [5,6]. The crucial role of the correct enantiochemistry can be also seen in the fact that organisms tightly control the use of D-amino acids. Some of the aminoacyl-tRNA synthetases, which implement the genetic code by loading a tRNA with the corresponding amino acid have proofreading capabilities to exclude D-amino acids [7]. Additionally, organisms have evolved specific enzymes, deacylases, which are able to recognize tRNAs acylated with a D-amino acid and cleave the bond between the amino acid and the tRNA, thus preventing a D-acylated tRNA from entering into protein synthesis [8].

Another type of asymmetry is encountered in the conformation of the peptide bond connecting the carboxy end of one amino acid to the amino end of the next one in a peptide or protein. Due to the partial double-bond character of the C_*n*_-N_*n*+1 _bond, the atoms C_*α*, *n*_, C_*n*_, O_n_, C_*α*, *n*+1_, N_*n*+1 _and its hydrogen are in a plane (see, however, Ref [9]) and the rotation around the C_*n*_-N_*n*+1 _bond is restricted by a barrier of about 20 kcal/mol [10,11]. Depending on the value of the dihedral angle *ω *described by C_*α*, *n*_, C_*n*_, N_*n*+1 _and C_*α*, *n*+1_, one can distinguish *cis *(*ω *≈ 0°) and *trans *(*ω *≈ 180°) isomers [12] (see Figure 1C). For sterical reasons, the *trans *isomer is energetically more stable and, thus, is the prevalent form in proteins. Additionally, the rather high rotational barrier makes the interconversion of the two isomers a very slow process at room temperature. Nevertheless, *cis *peptide bonds can be found in nature [13,14]. The vast majority of *cis *bonds are observed before a proline residue, Xaa-Pro, with Xaa being any amino acid. The formation of these *cis *peptides is catalyzed by special enzymes, prolyl-*cis*/*trans *isomerases [15,16]. Prolyl-*cis*/*trans *isomerization is an important molecular switch [17] and the occurrence of enzymes specialized for this particular isomerization underpins its biological significance. Non-prolyl *cis *peptide bonds can also be found in proteins, but much less frequently than Xaa-Pro [13,14] and only one protein, DnaK, is known to promote peptide isomerization of non-prolyl peptide bonds [18]. In particular, DnaK was found to accelerate the isomerization of Ala-Xaa bonds, Xaa being Ala, Gly, Glu, Ile, Leu, Lys, Met, or Ser. Metal ions can also play a role both in stabilizing the *cis *isomer and in promoting isomerization [19-22].

Correct stereochemistry of a structural model is important for its interpretation and critical if the model is to be subject to a molecular dynamics simulation. Force fields typically employed in biomolecular simulations do not contain terms to enforce stereochemistry and support either enantiomer or peptide isomer. Errors in the input structure usually persist throughout the simulation and, as will be shown below, can propagate and lead to severe artifacts. Even in cases where stereochemical errors do not lead to such large-scale problems, such errors must be avoided since they represent deviations of the simulated system from the biological reality that is to be modeled. There is a steady trend in the field of biomolecular simulations toward the study of large biomolecular assemblies and the usage of models based on structure prediction. Our recent experience [23-25] shows that stereochemical errors often arise in the preparation of large systems for simulation, particularly when some components must be modeled prior to simulation.

A variety of servers and programs are available for structure validation, a vital stage in the preparation of files for deposition in a coordinate database. One example is the SAVES server [26], which provides an interface to tools such as PROCHECK [27], WHAT_CHECK [28], and other programs to detect irregularities in geometry and structure such as chirality, bond angles, close contacts, or rotamer states of amino acid side chains. Other examples with similar functionality include the MolProbity [29] server as well as the PDB validation service [30].

All of the aforementioned tools for structure validation are primarily designed to validate experimentally obtained models and not to ensure proper stereochemistry in simulations. Additionally, although irregularities are reported, none of the tools we are aware of allow the user to inspect and correct stereochemical errors easily and immediately. We have thus written software tools to help researchers easily detect, correct, and avoid stereochemical errors in simulations.

In the following, we start by illustrating how errors in chirality and peptide bond configuration can affect secondary structure of proteins in molecular dynamics simulations. We then present software tools to identify, visually inspect, and interactively correct stereochemical errors in structural models of proteins and nucleic acids. Next, we discuss the most common sources of stereochemical errors in simulations, alongside a systematic analysis of the entire Protein Data Bank [31]. Finally, we provide a recommended workflow to avoid stereochemical errors in biomolecular simulations.

## Results and Discussion

### Consequences of stereochemical errors in biomolecular simulations

**Figure 2:**
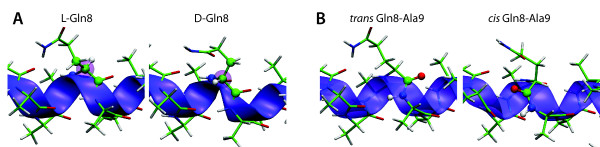
**Stereochemical manipulations used for the illustrative simulations**. Stereochemical manipulations used for the illustrative simulations. (A) Different configurations at the chiral C*_α _*at Gln8. (B) Different isomers at the Gln8-Ala9 peptide bond.

In order to understand more directly the effects that errors in chirality or peptide bond configuration have on secondary structure, consider three simulations involving a 15-amino-acid-long *α*-helix AAQAAAAQAAAAQAA solvated in water. In the first simulation, the helix is stereochemically correct. For the second simulation, a chirality error is introduced at C*_α _*of Gln8 (Figure 2A). In the third case, the peptide bond between Gln8 and Ala9 is present as a *cis *isomer (Figure 2B).

**Figure 3:**
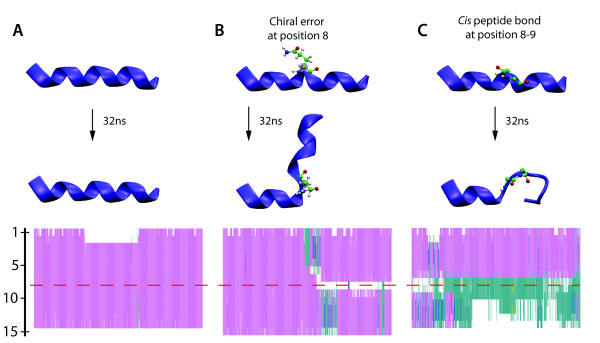
**Impact of stereochemical errors on the structure of an *α*-helix**. Impact of stereochemical errors on the structure of an *α*-helix. The figure shows the starting conformations, the conformations after 32 ns of equilibrium simulation, as well as the secondary structure content throughout the simulation for (A) a stereochemically correct helix, (B) a helix with a D-Gln8, and (C) a helix with a *cis *peptide Gln8-Ala9. The axis on the left shows residue numbers and the red dashed line indicates the position of the introduced stereochemical errors. The colors in the bottom panels represent secondary structure content: helix (pink), turn (cyan) and coil (white).

Figure 3 shows the conformations of the peptides after 32ns for each of the three scenarios, as well as the secondary structure content throughout each trajectory. By the end of the simulation the stereochemically correct helix is intact; in fact, the secondary structure is maintained during the entire simulation (Figure 3A). In contrast, a flip in chirality introduces a kink of almost 90° into the helix (Figure 3B). Interestingly, the two pieces separated by the kink remain helical. This conformational change can be understood considering that a D-amino acid in an environment with the backbone dihedrals *φ*/*ψ *of an L-amino acid helix features unfavourable steric interaction between the side chain and the peptide oxygen atom. The relaxation leading to the very prominent kink brings the side chain of Gln8 out of the eclipsed conformation relative to the peptide oxygen atom. In case of the *cis *peptide bond, the initial disturbance in the hydrogen bond network stabilizing the helix leads to a rearrangement of the network and to a complete loss of helicity downstream of Gln8 (Figure 3C). The configuration of a peptide bond is central to the types of secondary structure the peptide chain can assume: only the *trans *isomer accepts and donates hydrogen bonds in opposite directions allowing for formation of *α*-helices and *β*-sheets.

As demonstrated above, the impact of errors in chirality or peptide bond isomerization on secondary structure can be dramatic. Note, however, that the chosen example represents the worst case scenario in terms of severity of the structural disturbance - in a real protein, tertiary interactions may provide stabilization of the native structure and, thus, dampen the effect or extend the time scale on which the structural disturbance becomes apparent.

### Tools to identify, inspect, and correct stereochemical errors

Having demonstrated the impact of stereochemical errors on structure, the question of how to ensure stereochemical correctness in simulation naturally arises. In general, biological systems can contain amino acids or sugars of different chirality, as well as both peptide isomers. Thus, an automatic procedure to "correct" the structure is not appropriate unless one is absolutely certain that only one enantiomer and isomer occurs. Therefore, we designed a semi-automatic four-step protocol to correct errors and to ensure stereochemical integrity of a simulated system. For both chirality and peptide bond conformation, the protocol was implemented into easy-to-use plugins for the molecular visualization and analysis program VMD [32], referred to as Chirality and Cispeptide plugins, respectively. The plugins make use of the molecular dynamics simulation package NAMD [33] in the correction step. Both software packages are open source and freely available. The current implementation provides both a graphical and a command-line user interface. Use of each plugin follows a similar 4-step process, namely: (1) identify stereochemical anomalies; (2) visually inspect each anomaly and decide if it should be corrected; (3) move selected atoms as to change the stereochemical configuration; and (4) locally optimize the structure. In the following, we describe the four steps using the Cispeptide plugin as an example. Full documentation is available on the VMD website [34]. An additional step-by-step practical guide can be found in a tutorial describing both plugins [35].

**Figure 4:**
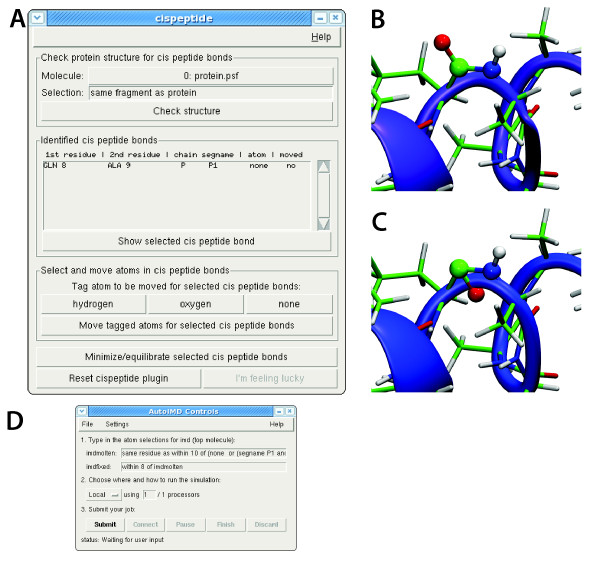
**The ****Cispeptide****plugin**. (A) The main window of the plugin showing the selection options in the top form, identified *cis *peptide bonds in the center form, and options to move atoms and to relax the structure in the bottom form. (B) Molecular visualization highlighting the selected bond generated by the plugin. (C) The configuration generated upon moving the oxygen atom along the C-O bond. (D) The control window for interactive molecular dynamics used in the correction of unusual configurations.

In step (1), unusual stereochemical configurations (*cis *peptide bonds in case of the Cispeptide plugin) are identified. A peptide bond is considered to be an irregularity if the value of the dihedral angle *ω' *formed by O_*n*_, C_*n*_, N_*n*+1 _and C_*α*, *n*+1 _is larger than 85°. The slightly different, but equivalent, definition of the angle with respect to *ω *ensures unique atom names used for the angle measurement (exploited for computational efficiency) and the threshold is chosen so as to avoid false positives while maximizing the sensitivity when all the structures of the PDB were tested (see "Sources of stereochemical error below"). For chiral centers, the improper angle made up of non-hydrogen atoms with the chiral atom in the center is used to define non-standard configurations: a negative value of the considered improper indicates an unusual chirality. All identified irregularities are displayed in the corresponding panel (see "Identified cis peptide bonds" in Figure 4A). In step (2), the irregularities are inspected and a decision is made whether to keep or to modify each stereochemical configuration. Clicking on each stereochemical anomaly generates a corresponding molecular visualization (Figure 4B). As mentioned before, there are tools [36] that may help decide if the *cis *configuration is correct or if it should be switched to *trans*. In step (3), for each *cis *peptide bond to be manipulated, the user selects an atom (hydrogen or oxygen) to be moved (see Figure 4A). C*_α _*atoms are not allowed to move in order to minimize the structural impact. The movements are reflections of the positions of hydrogen or oxygen relative to the peptide nitrogen or carbon, respectively (see Figure 4C). Which of the two atoms should be moved highly depends on the environment, and is thus not automated. By visual inspection, the user can quite confidently determine which atom (hydrogen or oxygen) should be moved in order to optimize the hydrogen bonding network. Finally, in step (4) a local structure optimization is carried out using NAMD through the interactive molecular dynamics [37,38] interface in VMD (Figure 4D). To enforce the target isomer, local restraints on the peptide dihedral are employed. The local nature of the manipulation and relaxation, which only involves residues within a given cutoff, prevents widespread influence of the procedure on the system. Finally, once the system is stereochemically correct, restraints can be constructed and used during further relaxation steps, prior to production simulations, to prevent changes in stereochemistry.

### Sources of stereochemical errors

Most molecular dynamics simulations of single proteins are relatively safe from stereochemical errors, given that molecular structures are validated upon deposition into the PDB. The irregularities detected in the validation step are usually either corrected or reported in the header of the PDB file. Using a structure from the PDB for simulations, together with a careful examination of the file header, usually means that the stereochemical integrity of the simulation is secure (barring special cases discussed below), because during an equilibrium simulation the force field will preserve stereochemistry. However, since force fields used for molecular dynamics simulations support both types of chirality as well as both peptide bond isomers, an existing error will persist throughout the simulation.

To quantify stereochemical anomalies within the Protein Data Bank, all the structures contained in the PDB were analyzed using the Chirality and Cispeptide VMD plugins introduced above. As shown in Table 1 around 4,000 unusual configurations with respect to chirality could be found in protein and nucleic acid structures, as well as more than 100,000 *cis *peptide bonds. Analysis of the complete dataset shows that one chirality error is expected for every 10,000 residues, whereas for every few hundred residues a *cis *peptide bond can be expected. The likelihood of stereochemical anomalies varies with the structure determination method, in particular when chirality is concerned. For example, for structures derived from electron microscopy, one chirality error can be expected for every 500 residues (see Table 1). The higher error rate seen in electron microscopy-derived structures are likely due to the much lower resolution on average, combined with use of a wide range of modeling approaches, including manual manipulation of structures.

**Table 1:** Chirality errors and cis peptide bonds in the Protein Data Bank

Method^1^	Chirality errors				*Cis *peptide bonds			
	
	Total^2^	Residues per error^3^	**Structures analyzed**^**4**^	Structures with errors	Total^2^	Residues per *cis *bond^3^	**Structures analyzed**^**4**^	Structures with *cis *bonds
All	4047	9746	67942	648 (1%)	104455	368	65899	28576 (43%)
Electron microscopy	1273	529	201	40 (20%)	2316	262	192	93 (48%)
Solution NMR	280	2684	8444	105 (1%)	1289	565	7574	818 (11%)
X-ray diffraction	2494	15230	59147	503 (1%)	100776	368	57987	27646 (48%)

^1 ^As given by EXPDTA record on each PDB file. Entries with more than one method were classified based on the first method listed. Only methods with at least 100 solved structures are shown in the table.

2 Total number of chirality errors or *cis *peptide bonds detected by the Chirality and Cispeptide VMD plugins, respectively.

^3 ^Total number of residues divided by total number of chirality errors or *cis *peptide bonds. For chirality errors, all protein and nucleic acid residues are counted; for *cis *peptide bonds, only protein residues are counted.

^4 ^For chirality, only structures containing at least one protein or nucleic acid residue were considered. For *cis *peptide bonds, only structures containing at least one peptide bond were kept in the analysis.

In a subset of structures, chirality errors (153 structures) and *cis *peptide bonds (62 structures) were reported in the PDB file, but were not detected by the plugins. To understand the discrepancies, about half of the structures in each subset was visually inspected. In the case of *cis *peptides, the differences were due to missing atoms in the deposited model, errors being present in only one alternative conformation or model (see Methods), pathologically distorted structures, and, most frequently, incorrect entries in the PDB header. Similarly, the reasons for the discrepancies in chirality were that errors occurred in cofactors not checked by the plugin, the anomalies were present in only one alternative conformation or model (see Methods), or the annotations in the PDB header were incorrect.

The plugins also identified many structures with stereochemical anomalies not reported in the PDB header. According to the current PDB format standard, each *cis *peptide bond should be reported in a separate CISPEP record. It is then possible to compare each identified *cis *peptide bond with the PDB header. There were in total 2,518 *cis *peptide bonds identified by the Cispeptide plugin but not reported in the PDB files. Visual inspection of 20 such files did not reveal any incorrect identification by the plugin. Chirality issues should be reported in CAVEAT records of the PDB header. Unfortunately, since CAVEAT records are free format, rarely are chirality errors individually reported. Furthermore, many PDB structures have chiral errors inconsistently documented in REMARK 500 records instead. Thus, a similar comparison on a per-error basis cannot be performed for the Chirality plugin; instead, we can only report that 348 PDB files contain chirality anomalies according to the Chirality plugin, but lack the corresponding annotation in their PDB headers. Upon visual inspection of 20 files, the structures fell into two categories: either there should be a D-amino acid (non-ribosomal peptides) or the PDB header is simply missing the required annotation.

Apart from errors already present in experimentally determined structures, our experience shows that there are three main sources of stereochemical errors in simulations. The first source is found in modeling steps, particularly homology modeling: any regions of a structure that were modeled *de novo*, especially at the junctions of the known and modeled part of a protein, are prone to peptide isomerization errors. The second common source is found in the setup protocol for a simulation. This includes the preparation of the system and its initial relaxation. In particular, sterical clashes between atoms can lead to errors in chirality and isomerization state of peptide bonds. Again, such behavior is particularly likely at the interface between known and modeled portions of a structure, which may contain severe distortions prior to equilibration. Although the barriers for isomerization or a flip in chirality are large enough to prevent these events in an equilibrium simulation at physiological temperatures (e.g., 21 kcal/mol in CHARMM22 [39]), forces arising during the initial structure optimization, necessary to relax possible clashes, may be large enough to introduce errors into the structure. This source of errors becomes increasingly important as the field of computational biophysics moves towards multi-component assemblies, the structures of which are often modeled based on high-resolution models of their constituents and low-resolution data of the whole complex. Finally, errors can also be introduced during structure optimizations if additional forces are applied on the system. Of particular interest at this point is the molecular dynamics flexible fitting (MDFF) method [40], which flexibly fits atomic-resolution structures into low-resolution density maps. In some (rare) cases, it was observed that forces arising from the MDFF method during initial structure optimization were large enough to cause stereochemical errors. System setup protocols where the system is simulated at very high temperatures (e.g., to obtain heat-denatured structures) may also allow incorrect isomerization events.

The Chirality and Cispeptide plugins can be used to generate harmonic restraints designed to preserve the current isomerization state of each chiral center and peptide bond. The restraints can be used in simulations with NAMD, effectively preventing stereochemical errors from arising during simulation. These restraints should be removed prior to production equilibrium simulations, since they are not required and would represent an unnecessary modification of the force field employed.

### Recommended workflow

In order to avoid stereochemical errors, the following simple workflow is recommended for standard MD simulations:

1. Build system for MD simulation (model missing components, assemble structure, embed system in a water box, and add counterions).

2. Energy-minimize structure.

3. Check stereochemistry with the Chirality and Cispeptide plugins, correcting errors if applicable. Repeat until no further errors are detected. Make sure that the detected irregularities are indeed errors and not naturally occurring.

4. Proceed to production simulation.

For simulations in which large forces are expected (e.g., flexible fitting with the MDFF method [40] or temperature-induced denaturation), it is recommended that, in addition to the workflow above, harmonic restraints generated by the Chirality and Cispeptide plugins are applied throughout the simulation.

## Conclusions

The simulations presented here illustrate the drastic effects that stereochemical errors can have in biomolecular simulations. Experimentally determined structures may contain stereochemical errors, and various modeling approaches can further increase the number of such errors. As the community moves toward simulation of large, multi-component complexes and uses to an increasing extent models based on structure prediction, the issue of stereochemical correctness becomes even more relevant. We thus developed tools to identify, inspect, and correct stereochemical errors in protein and nucleic acid structures. In particular, chirality and the isomerization state of a peptide bond are examined. The main advantage of the offered tools is the possibility to immediately inspect and correct the detected errors. The tools are implemented as plugins to the molecular visualization and analysis program VMD. The recommended workflow presented above effectively avoids artifacts in simulations due to stereochemical errors. We hope that checks for stereochemical correctness become a standard step of any biomolecular simulation or generation of predicted structural models for proteins and nucleic acids.

## Availability and Requirements

• Project name: cispeptide, chirality; included into VMD

• Project home page: http://www.ks.uiuc.edu/Research/vmd

• Operating system(s): Platform independent

• Programming language: Tcl

• Other requirements: VMD 1.9 or higher, for molecular dynamics part: NAMD 2.7 or higher

• License: UIUC Open Source License http://www.ks.uiuc.edu/Research/vmd/plugins/pluginlicense.html http://www.ks.uiuc.edu/Research/namd/license.html

## Methods

### Molecular dynamics simulations

Molecular dynamics simulations were conducted using NAMD 2.7 [33]. The system consisted of the 15-amino-acid-long *α*-helix AAQAAAAQAAAAQAA solvated in TIP3P water. The *N*- and *C*-terminus were acetylated and amidated, respectively. The system was set up in VMD [32]. In particular, the helix was constructed using the molefacture plugin, after which the full system was built with the solvate and psfgen plugins.

All simulations were performed in the NpT ensemble (*T *= 310 K, *p *= 1 atm). The stereochemically correct system was equilibrated using a 2-step protocol. First, water and side chains were equilibrated for 400 ps while the backbone of the peptide was restrained, after which all restraints were removed and the system was equilibrated for an additional 3.6 ns. The resulting structure was used to manually introduce a chirality flip at Gln8 and to isomerize the peptide bond between Gln8 and Ala9 into the *cis *form (see Figure 2). Starting from the three obtained systems, the simulations were continued for 32 ns in each case. The equations of motion were integrated using periodic boundary conditions and a 2-fs time step, with bonded interactions calculated every 2 fs. Nonbonded, short-range interactions were calculated every 4 fs using a distance cut-off of 10 Å with a switching function applied at 9 Å. Long-range electrostatic interactions were updated every 6 fs using the Particle Mesh Ewald (PME) method and the PME grid density was never less than 1/Å^3^. The simulations presented here were performed with the CHARMM27 force field [39,41] with the CMAP correction [42].

### Analysis of the PDB

Each structure available in the PDB as of mid-September 2010 was analyzed using the Chirality and Cispeptide VMD plugins. In case of multiple models (typically in structures solved by NMR), only the first model was considered in the analysis. When multiple alternative conformations of certain residues were present, as indicated by the altLoc field in the PDB file, only the first conformation was considered. For comparison and validation purposes, each PDB file header was parsed for reported *cis *peptide bonds (CISPEP records) and unusual chirality configurations (CAVEAT and REMARK 500 records).

## Authors' contributions

ES and LGT conceived the ideas behind the paper. LGT wrote and tested the software. ES assisted in writing and testing of the software. PLF assisted in testing the software. ES performed and analyzed molecular dynamics simulations. LGT analyzed structures of the Protein Data Bank. All authors were involved in writing of the manuscript. All authors read and approved the final manuscript.
